# Strategies to Cope With the COVID-Related Deaths Among Family Members

**DOI:** 10.3389/fpsyt.2021.622850

**Published:** 2021-02-15

**Authors:** Lidia Borghi, Julia Menichetti

**Affiliations:** ^1^Clinical Psychology, Department of Health Sciences, University of Milan, Milan, Italy; ^2^Institute of Clinical Medicine, University of Oslo, Oslo, Norway

**Keywords:** COVID-19, grief, coping strategies, loss management, family bereavement

## Abstract

The extraordinary circumstances of deaths during COVID-19 pandemic have been challenging for the deceased's families. This contribution aims to describe some spontaneous strategies that family members may adopt to cope with the loss of a relative for COVID-19. The present reflection derives from the experience of a clinical psychology unit of one of the biggest public hospital in Milan, Italy, which supported 246 families of COVID-19 victims in the 1st days after the loss. Spontaneous strategies used by family members to deal with such a unique mourning process involved: creating alternative good-bye rituals, normalizing the loss, addressing faith and hope, highlighting the perks of isolation, supporting others in need, and delivering the bad news to others. These observed strategies may suggest how to assess and support a “normal” bereavement process during the extraordinary COVID-19 circumstances, in order to prevent further psychological distress.

## Introduction

During the COronaVIrus Disease 2019 (COVID-19) pandemic, especially in the emergency phases, people have been dying in extraordinary circumstances, which have been affecting relatives' grieving process (1), especially for people who lost a relative at the hospital (2). In fact, safety measurements and restrictions often prevented physical closeness: families could not stay close to the loved one in the last moments of life (3, 4). Moreover, some people have been forced to stay at home after the loss to prevent the spread of the virus, reducing the possibility of support from their social network (3, 4). Also, traditional funeral ceremonies - which usually have an important role in the grieving and recovery process, as they foster emotions' expression, social support, and give meaning to the loss (5) - have been banned or limited (6).

The complexity and simultaneity of these stressful and limiting conditions can make the grieving process extremely challenging for families (7); therefore, the possibility of processing the loss of the deceased has been at risk (3, 4). Furthermore, the support usually provided by healthcare professionals has been limited itself by COVID-19 safety restrictions, a small amount of time and little available resources (3, 8). In such a unique scenario, how can families process the loss of a relative for COVID-19? Which spontaneous strategies emerged in dealing with this extraordinary grief?

The current perspective article aims to reflect on these questions, by presenting a secondary analysis of data collected for another research study focused on reporting the experience of a clinical psychology unit of one of the biggest public hospital in Milan, Italy, which, during the first wave of the pandemic, supported 246 families of COVID-19 victims who died at the hospital. This psychological support was delivered through a phone follow up made by hospital psychologists 2–3 days after the communication of the loss. The contents, the procedures, and the specific aim of these interventions are reported elsewhere, along with details about methodological and ethical procedures (9, 10) (with ethical approval gained in advance of collecting the data). In the present perspective article, we used this clinical experience and research data (in particular, the one derived from written reports that psychologists filled after each call) as the basis to inductively extract recurrently strategies that family members spontaneously showed to cope with the loss in the very first moments after the loss. In the conclusion, we reflect on them and on how these strategies may be useful to help other people in a similar situation.

## Families' Spontaneous Strategies to Cope With COVID-19 Deaths

From the clinical and research experience that the hospital collected during the first wave of the pandemic in Milan, Italy (9, 10), we extracted the main strategies that families reported 2–3 days after the loss of a loved one at the hospital as their way to cope with the bereavement. In particular, families often reported the following six behaviors and thoughts: finding alternative rituals; normalizing the loss; addressing faith and hope; addressing the positive sides of the isolation; supporting others in need; and communicating the bad news. Such strategies are reported below in detail and connected to relevant literature if there is any.

### “Floating Deaths”: Creating Alternative Rituals for Grieving

Physical distance and isolation could lead to a detached experience, transforming the loss in a “floating death,” not truly realized, and therefore not processed. Nevertheless, our clinical experience with bereaved families showed that those lonely funerals and limited social rituals did not become “missing” funerals. As a matter of fact, similarly to what happened with the Ebola disease outbreaks (11), families found new ways to say the last goodbye and to celebrate the dead: they created new rituals able to ease their pain. Undertakers had a key role to facilitate this process: families often asked them to drive under the family home on their way to the crematory. This way, the last goodbye to the loved one could take place at the window, with a word, a song, or a prayer. In other cases, the undertaker was asked to place special items in/on the coffin or to take a picture of the prepared body. Furthermore, when at least one family member was able to physically attend the funeral, the last goodbye sometimes became a digital experience: the relative shared videos or broadcast the funeral to family and friends. Prayers also became a shared digital experience: e.g., some rosaries and other prayers were said together with the hospital priest on a video-call. Overall, technologies - phone calls, video calls, messages – had a key role to help families not feeling segregated and alone. Moreover, the idea of a future visit to the grave -sooner or later- was something that gave a timeframe and a contingency to the limitations, and thus consistency to the death.

### “Death in Old Age”: Normalizing a Loss Lived Under Extraordinary Circumstances

When the COVID-19 victim was already sick and/or old, the thought of a quick death often alleviated the sufferance, as death was seen as the natural course of events. “He/she was old, sooner or later this had to happen” was a recurrent thought among relatives that “normalized” a death lived under extraordinary circumstances. Such a process of “normalization” has been highlighted by previous studies as helpful for bereaved families (12). Furthermore, especially families already expecting the loss (e.g., families of already sick and old patients) lived the period before the death as an opportunity to open their hearts to the loved one and say words they never said before. This limited regrets and facilitated acceptance, leading to “peaceful” grief. Sometimes, death was even seen as a relief from very painful conditions.

### “Religious and Existential Anchors”: Addressing Faith and Hope

Addressing faith or hopes, with thoughts like “it was destiny” or “I hope he is buried in a nice place,” helped to come to terms with the death. Faith and spirituality represented an inner anchorage for families searching for meanings in an unpredictable and uncontrollable situation as the COVID-19 pandemic. They managed to reframe a stressful situation into a larger, more benevolent system of meaning. Spiritual resources – like attending church ceremonies or talking to a priest – were often limited, but families relied on an inner faith as a way to positively cope with the loss. Similarly, addressing hope – for the loved one, for themselves, for other family members or friends, for the community and society – could be the first step of grieving.

This is consistent with the previous literature showing how the relative's spirituality and religiosity can represent an important individual protecting factor for normal mourning, giving meaning to the loss and facilitating a process of transformation and identity reconstruction (13, 14).

### “Away From the Normal Life”: More Time to Process the Loss

Besides its many downsides, the safety measures and confinement restrictions offered some advantages: for example, families had more time to process the bad news, because they did not need to come back to normality in a few days, as it usually happens. This prolonged “time and space” to digest the loss facilitated some aspects of the mourning process, such as reflecting on the loss and emotions processing. At the same time, the practicalities required by the extraordinary situation of the pandemic (e.g., prolonged bureaucracy) offered an occasion to sustain the natural process of shifting between facing the painful reality and avoiding it by doing something else, as proposed by the dual-process model of coping with grief (15).

### “Helpfulness”: Supporting Others, Supporting the Self

The death of a dear one for COVID-19 was usually a piece of a larger problematic scenario in which stressors piled up. For example, often there was more than one sick relative within the same family, who may have been hospitalized or dead because of Coronavirus. Some people struggled to contain the spreading of the virus among the whole family. This resulted in feelings of anxiety and distress, but also of helpfulness. For someone, indeed, the need to focus on other family members' physical or psychological health helped them feeling useful, fostering their sense of helpfulness, power, and self-confidence. This is aligned with previous studies on exchanging experiences and support within communities sharing some characteristics or life event, such as the online grief groups: the possibility to provide help and support to others helps to mobilize internal resources to cope with the loss (16).

### “Delivering the Bad News”: Finding Words, Finding Meanings

Relatives and close family members often had to be the ones delivering the bad news to others. This could be a difficult task to accomplish in the 1st days after the loss. At the same time, it seemed to help bereaved individuals to uncover the silence surrounding the death and start to process it. Indeed, choosing the words can become a constructive activity of meaning-making in the mourning process (17). Especially in the confused and disruptive COVID-19 circumstances, delivering the bad news helped families translating experiences into meaningful narrations. This is consistent with the meaning reconstruction theory, which conceives narration as an activity that allows to re-author an experience of loss and to elaborate the loss by reorganizing, deepening, or expanding one's own beliefs and self-narratives to embrace the reality (18).

## Discussion and Conclusions

In the COVID-19 situation, most of the instruments that psychologists know as helpful for patients and families dealing with a death in the hospital, like family involvement, physical presence, clear information, and preparation (19, 20) have been negated. COVID-19 deaths are solitary, without traditional rituals, often unexpectedly fast, without clear information or preparation, and with limited or interrupted communication bridges (10). Therefore, COVID-19 deaths at the hospital, especially during the emergency phases of the pandemic, when Italian people were in major lockdown, might be lived as “bad deaths,” affecting the intensity and the quality of family members' grief reactions (21, 22). In this scenario, the description of the spontaneous strategies used by family members to cope with the losses may represent a precious resource, as it can provide first indications of “normal” bereavement process during the extraordinary COVID-19 circumstances and how to support it, preventing further psychological distress (23, 24). Such strategies represent new and creative ways to activate and to organize substitutive rituals and actions for grieving. They are concrete steps full of symbolic value which give meaning at the loss, helping a positive adjustment. These strategies are aligned with those proposed by previous literature on grief reactions after natural or unnatural losses: finding alternative rituals (11); normalizing the loss (12); addressing faith and hope (13, 14); using the isolation and the bureaucracy to oscillate between confronting the painful reality and avoiding (15); providing support to others living a similar experience (16); and reconstructing meanings by narrating the loss to others (18). This provides indications for considering these strategies helpful beyond the specific cultural and religious backgrounds of family members.

Future clinical and research studies are needed to better understand the long term connection between these spontaneous strategies and the grief processes of bereaved families who lost a relative for COVID-19. In particular, research is needed to assess how much these strategies facilitate a good psychological adaptation to the loss and prevent complicated grief. With further evidence, the presence (or absence) of these spontaneous strategies could be used to easily and early assess markers for future maladaptive grief processes, as well as to orient and support the normal grieving process. Finally, these strategies, as they represent natural strategies used by family members to start coping with the special circumstances of the COVID-19 related losses, could be used in early psychological interventions to assess and support these families in a way that is aligned to their natural ways to cope with these losses and to their specific experiences, as suggested by other studies (19).

## Data Availability Statement

The raw data supporting the conclusions of this article will be made available by the authors, without undue reservation.

## Author Contributions

JM and LB conceived the idea, collected the data, analyzed the data, and wrote the manuscript.

## Conflict of Interest

The authors declare that the research was conducted in the absence of any commercial or financial relationships that could be construed as a potential conflict of interest.
